# Bridging circular economy business models and rebound effects: Mapping systemic associations with semantic network analysis

**DOI:** 10.1007/s44498-026-00110-3

**Published:** 2026-07-15

**Authors:** Daniel Guzzo, Daniela C. A. Pigosso

**Affiliations:** Department of Civil and Mechanical Engineering, Technical University of Denmark, Kongens Lyngby, Denmark

**Keywords:** Rebound effect, Circular economy, Business models, Absolute sustainability, Sustainable consumption

## Abstract

While the circular economy (CE) holds significant promise, its potential sustainability gains are often undermined by rebound effects (RE). The field has documented numerous instances of RE in CE business models (CEBM) but lacks a systematic map of the associations between specific business model configurations and rebound mechanisms (i.e., the economic and behavioral causal chains that lead to RE). This research addresses this gap by developing and applying an interpretation-based semantic network analysis linking CEBM patterns to rebound mechanisms via triggers and drivers. The three primary contributions are: (1) a first semantic network of the associations between CEBM patterns and rebound mechanisms, offering important structural insights; (2) a clear positioning of triggers and drivers as the essential, actionable links between CEBM and RE; and (3) an operational causal ontology and complementary analytical procedures to support RE analysis within sustainability transitions. The ontology, semantic network and complementary analytical procedures provide a foundational framework for further explanatory studies in the field and for designing reboundless business models.

**Supplementary Information:**

The online version contains supplementary material available at 10.1007/s44498-026-00110-3.

## Introduction

Circular Economy (CE) is a promising approach to decouple resource consumption from economic activity by systematically narrowing, slowing, and closing the materials and energy flows (Bocken et al., 2016; Geissdoerfer et al., 2017). Despite the strong uptake of CE by companies and governments (Corvellec et al., 2022; Schröder et al., 2019), its potential to enable human activity within planetary boundaries is hindered by rebound effects (RE) (Calisto Friant et al., 2020; Corvellec et al., 2022; Schröder et al., 2019). RE are defined as systemic responses to interventions that offset their potential sustainability gains (Hertwich, 2005; Lange et al., 2021).

RE are widely documented across several circular strategies, sectors, and regions: (i) imperfect substitution and re-spending offsetting gains in smartphone reuse (Makov & Vivanco, 2018), (ii) closed-loop systems igniting additional production and consumption (Zink & Geyer, 2017), (iii) repair and access-based consumption inducing budget and lifestyle shifts (Ottelin et al., 2020), (iv) closed resource loops removing financial disincentives for overproduction (Greer et al., 2021), (v) availability of shared goods increasing consumption (Warmington-Lundström & Laurenti, 2020), (vi) moral licensing and reckless behavior stemming from reduced ownership (Siderius & Poldner, 2021), and (vii) service-based business models leading to reduced product care (Ackermann & Tunn, 2024).

The increasing evidence of RE in Circular Economy Business Models (CEBM) hints at a fundamental connection with rebound mechanisms – the economic and behavioral causal chains that lead to RE (Guzzo & Pigosso, 2024; Guzzo, Walrave, et al., 2024; Metic & Pigosso, 2022; Whalen & Whalen, 2020). Nevertheless, most empirical studies in CEBM are often ex-post and case-specific, focus on individual or limited sets of rebound mechanisms, and do not explore the cause-and-effect structures that can explain RE occurrence (Guzzo, Walrave, et al., 2024; Pigosso, 2024). This fragmented evidence creates a critical analytical challenge for both researchers and practitioners. While CEBM may cause RE in different ways, there is no systematic way to identify which of the many potential mechanisms are relevant to a given business model. Therefore, this research asks the foundational question: “What are the connections that systematically associate CEBM patterns with the activation of rebound mechanisms?”.

To answer this question, the research was guided by three specific research goals (RGs): RG1 – Identify how to systematically link CEBM and rebound mechanisms; RG2 – Map the relationships between CEBM and rebound mechanisms; and RG 3 – Derive structural insights that can inform practitioners and researchers investigating RE in CEBM. These goals were achieved through an interpretation-based semantic network analysis operationalizing a causal ontology emerging from the state-of-the-art literature, a method well-suited for systematically mapping the propagation of change within a knowledge domain.

This process began by reviewing the core knowledge related to CEBM implementation and rebound mechanism identification (Sect. 2). Next, a methodology was defined to systematically examine the relationship between these two areas (Sect. 3). We then introduce the ontology established in the study and detail the consolidated semantic network, offering important structural and practical insights (Sect. 4). Then, we discuss the implications of these findings (Sect. 5) and conclude with final remarks (Sect. 6). In a nutshell, this research advances proactive early-stage reflection on potential rebound pathways while supporting further explanatory research in the field.

## Research background

This section expands on the definitions and roles of CEBM patterns in design, justifying the choice of the set of patterns used in this research (Sect. 2.1). Then, rebound mechanisms, triggers and drivers are positioned as critical in associating RE and sustainability-oriented interventions (Sect. 2.2).

### Definitions and roles of Circular Economy Business Model (CEBM) patterns in design

CEBMs are configurations of value creation, value delivery, and value capture structures that deliver enhanced resource consumption (Geissdoerfer et al., 2018, 2020; Lüdeke-Freund et al., 2018; Pieroni et al., 2019) via operationalizing strategies like extending product use phases, intensifying asset utilization, cycling materials, or dematerializing solutions (Bocken et al., 2016; Geissdoerfer et al., 2017). After intensive effort in developing generic and sector-specific CEBM classifications that organize resource-effective strategies within the CE framework (Bocken et al., 2016; Ellen MacArthur Foundation, 2012; Guzzo et al., 2020; Moreno et al., 2016; Pieroni, McAloone, et al., 2020a, 2020b), the field has moved into shedding light on the recurring BM configuration options frequently employed by companies engaged in CE practice (Lüdeke-Freund et al., 2018; Pieroni et al., 2021). Overall, reaching configuration options helps minimize the complexity of design decisions and enables reaching CEBM concepts by assembling and configuration (Lüdeke-Freund et al., 2018; Pieroni et al., 2021).

Ludeke-Freund et al., (2018) performed a morphological analysis in the closed-loop supply chains and CEBM literature, reaching six CEBM patterns and a multidimensional morphological box that details the design options available, considering dimensions like value proposition and value delivery mechanisms. For example, the reuse and redistribution CEBM pattern combines take-back management services in the value proposition BM dimension and additional revenues from products for value capture. Pieroni et al., (2021) developed CEBM patterns relevant to manufacturing companies inductively from cases across multiple sectors, including electronics, furniture, machinery, medical devices, textiles, and food products. The patterns are organized into downstream (customer-facing) and upstream (operations-facing) value dimensions. Each pattern is deeply documented, providing details on the context, the resource problem addressed, the specific intervention logic, and exemplary cases. The source literature also provides prescriptive guidance on how to reach CEBM configurations, supported by complementary pattern cards that facilitate constructive discussions (Pieroni, Jensen, et al., 2020).

The CEBM patterns catalog and complementary pattern cards provide a clear structure and language, facilitating discussion at a suitable level for implementation. It also provides the most comprehensive, empirically supported consolidation of CEBM patterns in manufacturing to date. They are, therefore, good candidates for investigating the systematic association with other constructs, such as rebound mechanisms.

### Rebound mechanisms and the role of triggers and drivers

**Fig. 1:**
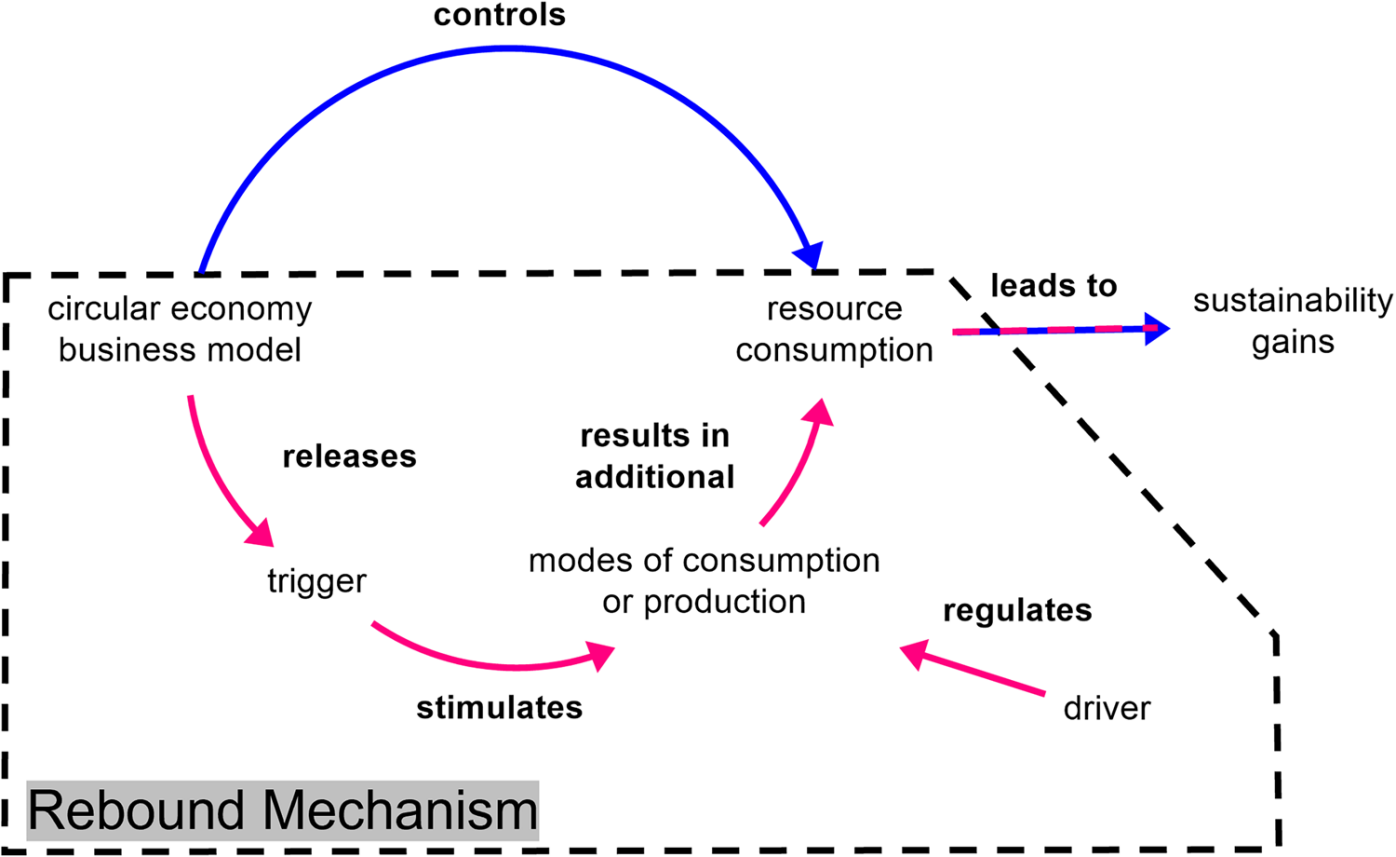
Conceptual framework for a rebound mechanism in CEBMs, building upon Guzzo et al., (2024)

By recognizing the importance of understanding RE causes (Font-Vivanco et al., 2016; Giampietro & Mayumi, 2018; Reimers et al., 2021; Sorrell, 2009), recent research has increasingly focused on the collection and categorization of rebound mechanisms (Colmenares et al., 2020; Guzzo, Walrave, et al., 2024; Lange et al., 2021; Metic & Pigosso, 2022). Rebound mechanisms are archetypal responses triggered by sustainability-oriented interventions (e.g., CEBMs) that limit potential sustainability gains. In that sense, while CEBMs aim to control resource consumption and contribute to sustainability gains, they simultaneously release triggers that stimulate additional consumption and production, thereby counteracting the potential sustainability gains (see blue and pink arrows in Fig. 1).

Understanding rebound mechanisms requires examining factors like financial, temporal, and moral resources (Sorrell et al., 2020) that induce RE (Colmenares et al., 2020). Similar factors can activate various rebound mechanisms, and their combined effect is not a straightforward sum of their individual effects (Gillingham et al., 2016; Lange et al., 2021; Madlener & Turner, 2016). Although there are indications of how different factors influence rebound mechanisms, the terminology used in the literature varies: terms such as "consumption and production factors," "determinants," "triggers," and "drivers" are often used interchangeably.

- Font Vivanco and van der Voet (2014) describe *consumption and production factors* as triggers of RE based on Hofstetter and Madjar (2003), arguing that "any change in these factors can lead to a RE".
- Vivanco et al. (2016) employ *drivers*, defining them as any economic factor that influences consumption or production activities and can be reasonably identified.
- Sorrell et al. (2020) employ *determinants* to denote the factors that explain economic and behavioral rebound mechanisms.
- Guzzo et al., (2024) employ *triggers* to represent the factors along the causal chain that explain a rebound mechanism, determining its existence.

Acknowledging complementarity in meaning, this research employs *triggers* to describe the factors that activate rebound mechanisms. Any change in the quantity, quality, or state of triggers may stimulate additional consumption or production activities, ultimately offsetting the innovation’s sustainability potential. Furthermore, certain factors moderate the relationship between trigger stimulation and increased resource use. For example, social and cultural values can influence consumer choices and moderate (i.e. as a driver) the effect of economic factors (i.e., trigger) on additional consumption (Font-Vivanco et al., 2016). This research employs *drivers* as regulators of the rebound mechanism, either amplifying or dampening the total RE (Guzzo, Walrave, et al., 2024).

While there is increasing clarity on the causal pathway that explains RE occurrence, a systematic mapping that links the design choices inherent in CEBMs to the specific rebound mechanisms they might activate remains absent from the literature. Establishing this foundation is crucial for effectively addressing RE.

## Methodology

This research employs an interpretation-based semantic network analysis (Doerfel, 1998) to operationalize a causal ontology (Lehmann et al., 1992) by analyzing state-of-the-art knowledge sets and synthesizing these associations using graphs and matrices towards a structured understanding of the associations between CEBM patterns and rebound mechanisms. This approach replaces common categorization-based methods that could show CEBM pattern and rebound mechanisms are linked but would not clarify their systematic relationship.

**Fig. 2:**
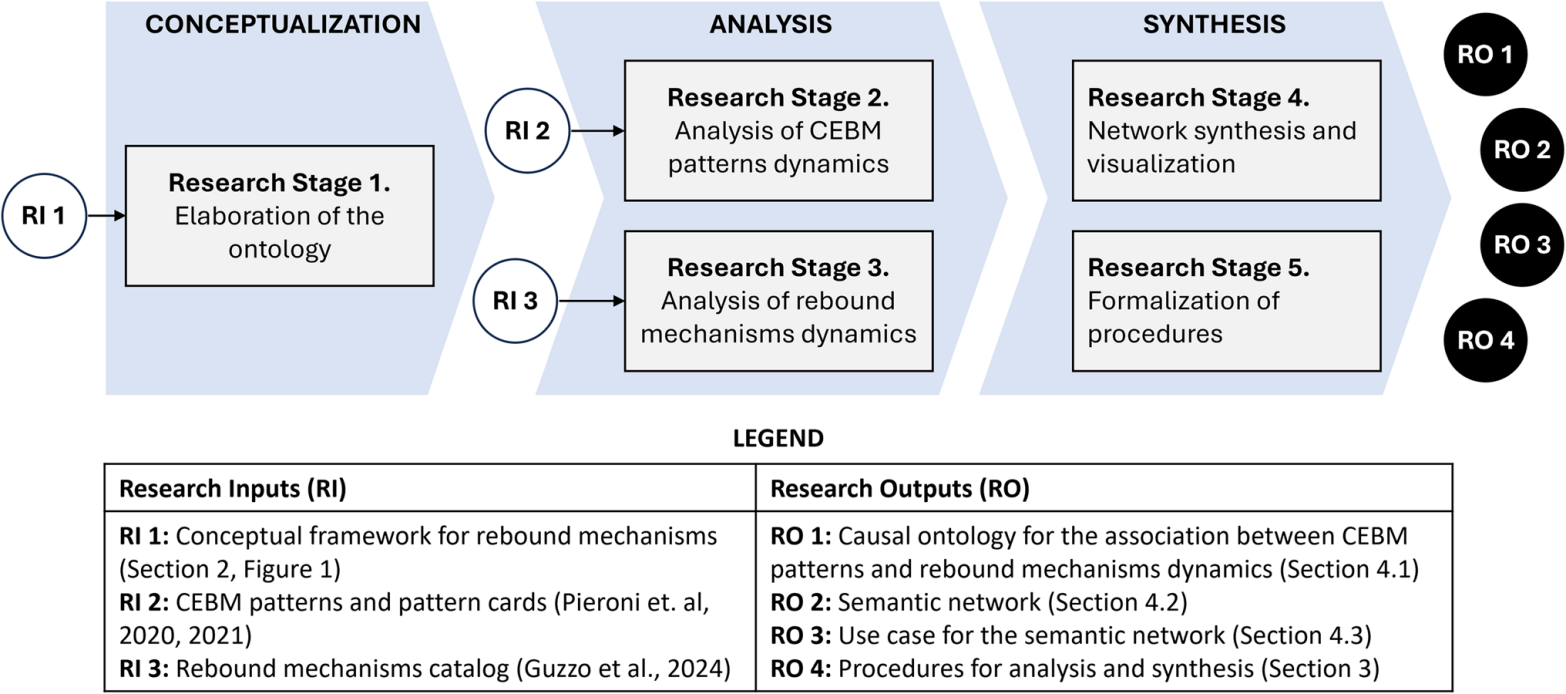
The four-stage research design, including research inputs and research outputs

The research followed five Research Stages (RS) organized into conceptualization, analysis, and synthesis and specifically builds on Research Inputs (RI) and results in Research Outputs (RO) – Fig. 2.

**Fig. 3:**
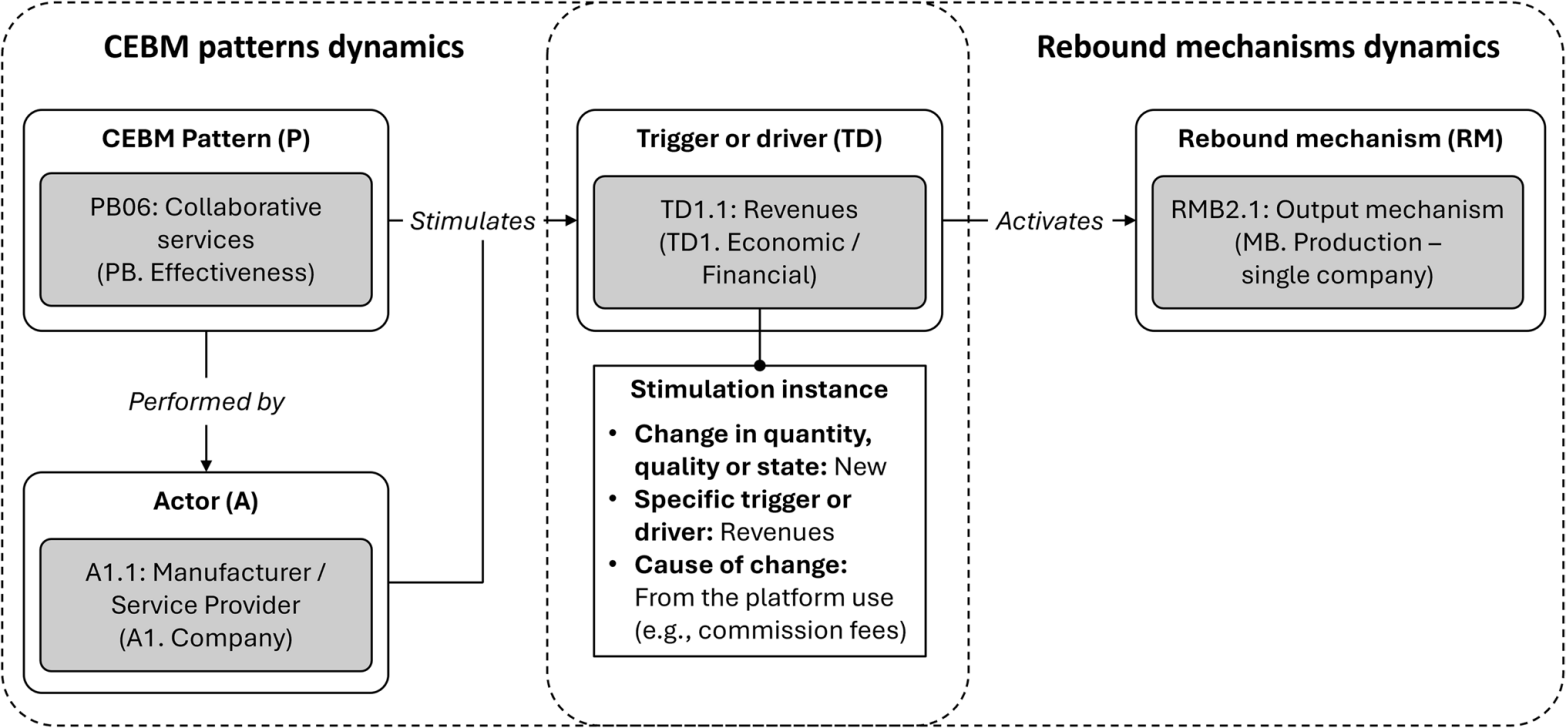
Causal ontology for associating CEBM patterns and rebound mechanisms through actors, triggers and drivers

**RS1: Elaboration of the ontology **established the study's theoretical structure. Departing from an existing conceptual framework for rebound mechanisms (**RI1**, Sec. 2, Fig. 1), we developed an operational causal ontology (**RO1**, Sec. 4.1, Fig. 3). A causal ontology determines the concepts, categories and relationships underlying the structure of meaning of a knowledge domain, while indicating the propagation of change (Lehmann et al., 1992). The ontology supports analyzes in RS2 and RS3 and is a primary research outcome. A categorized list of triggers and drivers from a literature review of fundamental RE papers (Guzzo et al., 2023) supports the ontology's applicability (**RO1**, Sec. 4.1, Table 1), ensuring a consistent analytical approach to study CEBM patterns and rebound mechanisms dynamics.

**Table 1:** Triggers and drivers explaining the stimulation of additional consumption and production activities

Type of trigger or driver	Definition	Triggers and drivers	References
Economic/ financial	Factors connected to money and material wealth that stimulate consumption and production activities	Price (and elasticity), consumption budget, household income, operational costs, profits, total cost of ownership	(Azevedo, 2014; Madlener & Alcott, 2009; Metic & Pigosso, 2022; van den Bergh, 2011)
Physical constraints	Factors influencing consumer and producer behavior related to generally constrained physical resources	Consumption time, production time, storage space	(Azevedo, 2014; Font-Vivanco et al., 2016; Metic & Pigosso, 2022; Santarius, 2016)
Consumer choice	Factors for individual behavior and decision-making that account for heuristics and biases	Preferences and perception, awareness of consumption intensity, environmental motivation, moral resources, moral hazard, habit, leisure time prioritization	(Azevedo, 2014; Font-Vivanco et al., 2016; Reimers et al., 2021; Sorrell et al., 2020)
Company choice	Factors connected to company decision-making to deliver business goals, such as shareholder value, and its overall vision and mission	Capital and labor productivity, reinvestment – in production factors, innovation, wages, supply adjustment	(Azevedo, 2014; Castro et al., 2022; Colmenares et al., 2020; Metic & Pigosso, 2022)
Socio-cultural influences	Societal and cultural factors influencing thoughts, feelings, behaviors, and consumption activities	Cultural acceptance, status, social feedback, social costs, market saturation	(Azevedo, 2014; Font-Vivanco et al., 2016; Sonnberger & Gross, 2018; Sorrell et al., 2020)
Goods’ attributes	The characteristics of products, services, and processes that influence the ability to differentiate, consume, or produce them, and determine their sustainability impacts	Substitutability, complementarity, utility/quality, number of functions, energy intensity, resource intensity, size of good, time intensity	(Azevedo, 2014; Gillingham et al., 2016; Metic & Pigosso, 2022; van den Bergh, 2011)

**RS2: Analysis of CEBM patterns dynamics** focused on populating the left-hand side of the ontology using qualitative content analysis (Elo & Kyngäs, 2008; Hsieh & Shannon, 2005), a systematic method for identifying the occurrence of concepts in text, while allowing for the subjective interpretation of the text’s context. This research focuses on the 24 industry-generic CEBM patterns for manufacturing companies and 34 complementary pattern cards (Pieroni et al., 2021; Pieroni, Jensen, et al., 2020) (**RI2**). The relationship between patterns and pattern cards is detailed in Table A-1 of Supplementary Information SI1. The analytical process involved two main steps:

**Step 2.1:** Pattern classification began by analyzing the causal chains within each pattern description. This involved systematically identifying the intervention, the specific changes in production and consumption dynamics, and their impact on resource consumption and production (see Table A-2, Table A-3, Figure A-1 of SI1 for the analysis of ‘*Collaborative services’* pattern, PB06). Following this, each CEBM pattern was categorized according to its primary strategic focus: (1) efficiency (i.e., optimized resource inflows or outflows); (2) effectiveness (i.e., enhanced value creation while keeping the resource inflows or outflows constant and (3) sufficiency (i.e., decreased need for value creation). Finally, (4) enabling patterns have an indirect contribution to other CEBM pattern(s), which enhances efficiency, effectiveness, or sufficiency.

**Step 2.2**: The systematic analysis of the CEBM patterns follows the sub-steps:

**2.2.1.** Identification of the changes caused by the CEBM pattern that could stimulate a trigger or a driver, facilitated by the list of triggers/drivers (**RO1**, Sec. 4.1, Table 1). Each stimulation instance was characterized into: (a) the specific change (e.g., an increase, decrease, or alteration in state); (b) the trigger/driver it represents (e.g., budget); and (c) its cause, as identified in the source text. Although exogenous, the analysis of drivers is relevant because they may hint at specific rebound mechanisms. For example, evidence on the substitutability between consumption options (e.g., between the shared option and other options) may indicate the potential for several types of indirect rebound mechanisms (for example, prioritizing shared cars over walking in a car-sharing case).

**2.2.2**. Attributing the stimulation instance to an ‘actor’ (i.e., any entity with a functional role in the system, including companies, individuals, or the good itself), which clarifies the domain where the rebound mechanism can occur.

**2.2.3.** The type of evidence was recorded for each identified stimulation (i.e., how close the claim is to the reference text): (1) explicit (directly stated in the text), (2) interpretive (inferred from the text's meaning and context), or (3) extrapolated (logically derived from a similar pattern or context – e.g., B2B instead of B2C).

For example, the texts describing collaborative services (PB06) indicate the pattern’s potential of removing “unnecessary costs… for the user”, understood as explicit evidence for potential release of budget (TD1.3) for renters (A2.1). This trigger stimulation instance was later extrapolated to company renters (A.1.2) as a potential decrease in operational costs (TD1.2) from fleet acquisition, by applying the pattern to a B2B context. Table A-4 of SI1 details all instances of triggers and drivers stimulation identified in PB06, categorized by actors and the type of evidence.

**RS3: Analysis of rebound mechanisms dynamics** focused on the state-of-the-art catalog of 26 mechanisms (**RI3**), selected for its comprehensiveness and explicit causal chains (Guzzo, Walrave, et al., 2024). Three redundant mechanisms were removed for clarity, as they were structurally similar and resulted in repetition. Since the catalog is based on the causal structure underlying the ontology used in this research, the analysis systematically deconstructed rebound mechanisms into triggers and drivers, assigning them to actor types aligned with the operational ontology. For example, the re-spending mechanism (M1D) was decomposed into the interplay between the cost of consumption (trigger) and the budget available (trigger) to individual consumers (actor), and the role of substitutability between consumption options (driver) and the resource intensity (driver); ultimately determining the RE of substituting away from more sustainable alternatives (Table A-5, SI1). This decomposition enabled operationalizing the connection of the rebound mechanisms catalog into the semantic network.

**RS4: Network synthesis and visualization** synthesized the analysis to represent the complete network of relationships. This involved both cross-tabulation using MS Excel PivotTables to develop relational matrices and identify the frequencies of associations between causally connected elements. Two visualization techniques were employed with a custom R script (10.11583/DTU.29528174), offering complementary abilities to display both granular causal logic and aggregate structures. Sankey diagrams were used to illustrate sequential causal relationships among the elements of the analytical framework. Chord diagrams were used to represent the interrelationships among element classes at a higher level of aggregation and to showcase all constructs' relationships at once. In both representations, the thickness of the flows indicates the frequency of connections identified in the knowledge sets. This process yielded two primary network representations: a consolidated semantic network (**RO2**) and a detailed use case using one CEBM pattern (**RO3**). Overall, the approach focuses on defining and examining the usefulness of the causal ontology and its structural insights rather than advanced network-analytic techniques.

Finally, **RS5: Formalization of research procedures** resulted in the procedures for analysis, synthesis, and demonstration (**RO4**), detailed in this section.

## Results

This section outlines the ontology and explicitly details the triggers and drivers identified in the literature (Sect. 4.1). Then, an overview of the consolidated network reveals critical structural insights (Sect. 4.2). Finally, the detailed use-case illustrates how the network can support decision-making in practice (Sect. 4.3).

### A causal ontology for the association between CEBM patterns and rebound mechanisms

The cornerstone of this research is the causal ontology, visually represented in Fig. 3. It illustrates the associations that underpin the analyzes, conceptualizing a two-stage logical association between CEBM patterns and rebound mechanisms (Fig. 3). First, in the CEBM patterns dynamics' domain, a CEBM pattern (P) is 'performed by' an actor (A) and 'stimulates' a trigger or driver (TD). Then, in the rebound mechanisms dynamics' domain, the trigger 'activates' a rebound mechanism (RM). The logic indicates that evidence for all associations suggests a potential RE.

A stimulation instance represents a specific analytical situation (grey background blocks) supported by the analysis of knowledge sets. For example, a manufacturer or service provider (A1.1) engaged in collaborative services (PB06) may generate new revenues (TD1.1) from platform use (e.g., via commission fees). In this case, they might use the profits from those revenues to increase production and counteract the potential sustainability gains from the CEBM (i.e., the output rebound mechanism, RMB2.1).

The categorized list of triggers and drivers that induce and/or moderate RE emerging from sustainability-oriented interventions (Table 1) is a critical constituent part of the ontology, as it enables both sides of the analysis. It is organized into six types: economic/financial, physical constraints, consumer choice, company choice, socio-cultural influences, and goods' attributes. Each type includes a definition and recurrent factors identified in the literature. For instance, "economic/financial" includes price elasticity, household income, and operational costs, while "consumer choice" covers preferences, environmental motivation, and habit. Most “goods’ attributes” may not directly stimulate consumption but can contribute to offsetting the sustainability gains. Notably, the list combines triggers and drivers as the same underlying factor may play a different role depending on the rebound mechanism involved. For example, while budget is a critical trigger for the income rebound mechanism, it can also be a driver of moral licensing by influencing perceptions of the effort required to act and, therefore, the accumulation of moral credits. Therefore, while the terms 'trigger' and 'driver' denote distinct functions in the rebound mechanism (activation vs. regulation), this taxonomy captures the underlying factors that can perform either role depending on the specific situation.

### Overview of the consolidated semantic network

**Fig. 4:**
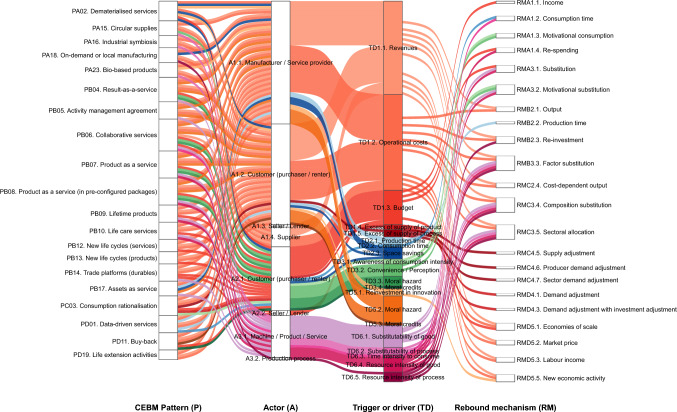
Sankey diagram synthesizing the association between CEBM patterns and rebound mechanisms dynamics within the semantic network. Elements are ordered hierarchically by class (e.g. PA to PD for Patterns). The flows representing the associations are color-coded by TD class: TD1 (Economic/Financial) in red, TD2 (Physical) in blue, TD3 (Consumer choices) in green, TD5 (Company choices) in orange, and TD6 (Goods attributes) in magenta. The data supporting Fig. 4 is available at https://doi.org/10.11583/DTU.29528174

Figure 4 demonstrates that the connections between CEBM patterns and rebound mechanisms are not limited to isolated instances but represent a systemic and pervasive feature of CEBM design. The analysis systematically charted 117 distinct instances of Trigger or Driver (TD) stimulation within CEBM dynamics, alongside 55 instances of Rebound Mechanism (RM) activation, considering those same TDs.

In the diagram, each block along an axis denotes a specific element in the taxonomy, while the flows represent unique causal pathways. A single flow tracing from left to right signifies a validated sequence in which a specific CEBM Pattern (P), performed by an Actor (A), stimulates a Trigger or a Driver (TD), which subsequently activates one or more Rebound Mechanisms (RM). Flow thickness is proportional to the frequency of unique causal connections identified in the knowledge sets. These flows are color-coded based on the specific TD they activate, highlighting their central role in bridging between CEBM patterns and rebound dynamics.

**Fig. 5:**
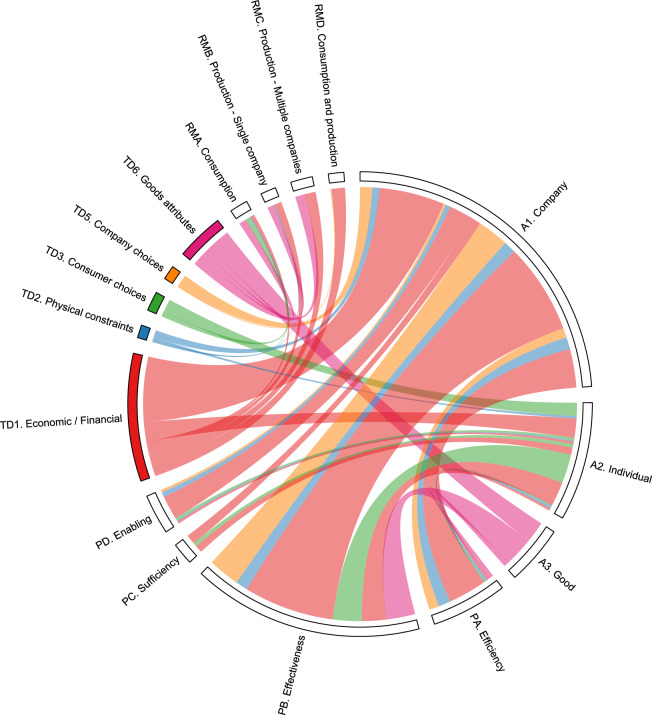
Chord diagram representing connections between classes of elements of the semantic network. The flows represent the volume of associations and are color-coded to match the TD classes identified: TD1 (Economic/Financial) in red, TD2 (Physical) in blue, TD3 (Consumer choices) in green, TD5 (Company choices) in orange, and TD6 (Goods attributes) in magenta. The data supporting Fig. 5 is available at https://doi.org/10.11583/DTU.29528174

A complementary chord diagram (Fig. 5) illustrates the extent to which these element classes interrelate at a higher level of aggregation, offering a holistic perspective on the structural interconnectivity between element classes. The complete description of the taxonomy elements is detailed in SI2. The frequency of associations between causally connected elements is available in the cross-tabulation matrices in SI3.

The analysis shows a high degree of connectivity at a pattern level: most CEBM patterns are highly connected, with 15 of 24 CEBM patterns linking to five or more distinct triggers and drivers (Table C-2, SI3). Crucially, this high connectivity transcends strategy types: 'effectiveness' patterns show the highest average number of connections (6.54), but 'efficiency' (5.2) and even the single 'sufficiency' pattern (6) also exhibit high connectivity (Table C-3, SI3). This demonstrates that the potential for RE occurs regardless of their primary strategic intent. Making this system of connections explicit provides the foundational means for proactively addressing RE by CEBM design.

The network architecture is dominated by economic logic, a bias partially explained by the CEBM patterns' focus on manufacturing in B2B scenarios. An intense concentration of connections exists around ‘Economic/Financial’ factors (TD1), which accounts for 60% of all identified connections between CEBM patterns and triggers/drivers (70/117), clearly exposing a bias towards value capture in the CEBM literature (Table C-2, SI3). This dominance persists to the other end of the chain: the network connecting triggers to mechanisms (Table C-6, SI3) shows the same 'Economic/Financial' factors (TD1) activate the highest number of unique rebound mechanisms (18). The most frequent individual triggers activating these mechanisms are financial: 'revenues' (TD1.1) and 'operational costs' (TD1.2). This analysis reveals that the common ground between the CEBM configuration and the activation of the rebound mechanism is mediated by economic logic, based on the current state of knowledge on both.

The network further reveals that the type of actor influences the frequency of trigger stimulation. The 'manufacturer/service provider' (A1.1) and the 'company as a customer' (A1.2) are the two most central nodes in the CEBM patterns and actors network, with 33 and 31 outgoing edges, respectively (Table C-1, SI3). There, the connection between 'companies' (A1) and 'economic/financial' factors (TD1) is strong, accounting for 54 of their 70 total outgoing edges. In contrast, links from 'individuals' (A2) show a more even distribution between economic factors (16 edges) and 'consumer choices' (11 edges) (Table C-4, SI3). This reveals that the CEBM patterns are oriented towards tangible financial benefits for corporate actors. At the same time, individuals are often targeted with intangible benefits, like flexibility and convenience, which are also well-known rebound triggers (Fig. 5).

Finally, the network exposes critical structural gaps and disconnections between the two bodies of literature. First, the analysis reveals internal disconnections, where several 'enabling' CEBM patterns (e.g., PD20-PD22, PD24) do not appear in the diagram. While their descriptions frame them as reinforcing other patterns (e.g., PD22: Own recycling reinforcing PA15: Circular supplies), their direct influence on triggers is not articulated in the source texts. Second, the map hints at isolated nodes between CEBM patterns and the dynamics of rebound mechanisms. Triggers identified in the CEBM descriptions (e.g., TD2.3: 'space savings’ and TD3.3: ‘moral hazard’) have no corresponding mechanism in the rebound catalog. Conversely, crucial factors known to explain rebound, such as TD1.4: 'excess of supply,' were absent from the CEBM pattern descriptions. Another notable finding is the lack of triggers for the 'Socio-cultural influences' (TD4) categories, suggesting that associations between the two fields still need to be established.

### A use case for the semantic network: analyzing the ‘Collaborative Services’ CEBM pattern

**Fig. 6:**
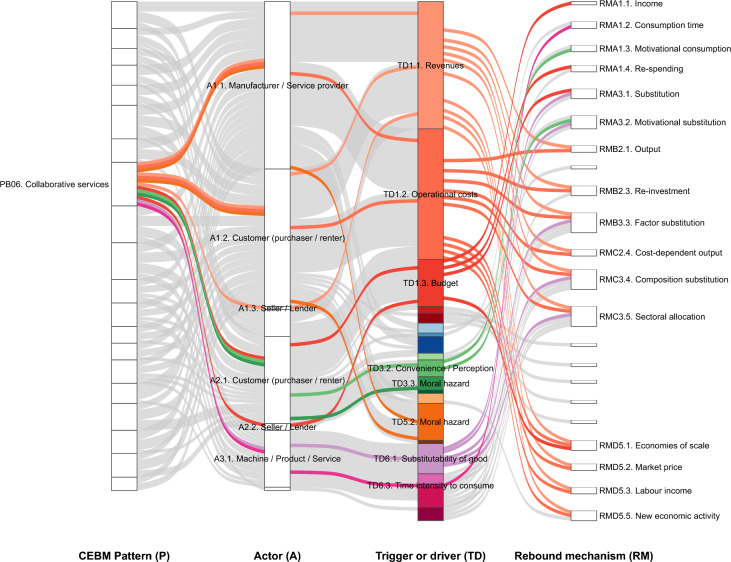
Sankey diagram showcasing the semantic network relevant to the CEBM pattern PB06: Collaborative services. The data supporting Fig. 6 is available at https://doi.org/10.11583/DTU.29528174

The semantic network highlights pattern-specific rebound pathways that may be relevant to companies designing or enhancing their CEBM. Figure 6 displays the elements that may be relevant when investigating the CEBM pattern PB06: Collaborative services. In a hypothetical B2C scenario, the service provider (A1.1) may generate additional revenues (TD1.1) through platform use. This can lead to several rebound mechanisms, including output (RMB2.1), factor substitution (RMB3.3), and economies of scale (RMD5.1). Concurrently, individual renters (A2.1) may benefit from increased convenience (TD3.2) due to the flexibility in addressing temporary product needs, which can potentially activate motivational consumption (RMA1.3) and motivational substitution (RMA3.2). Furthermore, reduced product access costs can result in additional budget (TD1.3), which may activate several mechanisms. With this information in hand, companies can devise strategies to address those triggers and tackle RE by design.

## Discussion

In this section, we elaborate on the implications of our findings, building on three main arguments. We first discuss the practical implications of exposing these pathways (Sect. 5.1). We then examine the biases revealed by our analysis (Sect. 5.2). Finally, we showcase how this research expands the understanding of RE (Sect. 5.3) and frame it as foundational for advancing RE research in CE and beyond (Sect. 5.4). Five critical limitations of this study are outlined, highlighting possible directions for future research.

### Turning the CE-rebound pathways visible and actionable advances rebound prevention

The evidence of causal associations between CEBM patterns and rebound mechanisms challenges the view of RE as an occasional side-effect of poorly designed models. Instead, it suggests that RE is an inherent structural property of CEBMs. The analysis in this research highlights that the very economic and social benefits driving CE adoption, such as reduced costs, new markets, or employment opportunities, are often the same triggers activating RE (Corvellec et al., 2022; Korhonen et al., 2018). The semantic network presented in this work makes such systemic associations explicit and can also serve as a practical guide for proactive early-stage reflection on potential rebound pathways.

Furthermore, the application of the proposed causal ontology clarifies the zone of influence for RE by attributing the stimulation of triggers and drivers to specific value chain actors, helping resolve the RE puzzle in CE (Ferrante et al., 2024). This distinction is critical for developing appropriate prevention strategies, as influencing an internal design choice is fundamentally different from shaping the behavior of clients and suppliers. By making these zones explicit, our work responds to calls for more systemic governance where multiple stakeholders coordinate actions to tackle RE (Castro et al., 2022; Schultz et al., 2024).

The network can function as a structured diagnostic tool to guide practitioners through a systematic inquiry, providing a transparent, question-led process for analyzing CEBM configurations and their context. It helps assess the most likely triggers and potential rebound mechanisms, ultimately pinpointing points of influence where prevention strategies can be applied. It offers a systematic and replicable path for exploring the complex web of actors and practices contributing to RE emergence (Niero et al., 2021).

Therefore, this diagnostic approach contributes to the growing research on actionable tools to address RE in product-service systems (Guzzo & Pigosso, 2024; Kjaer et al., 2019; Sarancic et al., 2023), CE business model experimentation (Das et al., 2023; Müller & Remke, 2025), and firm governance (Schultz et al., 2024; Zerbino, 2022). It stands out by providing an explicit logic for connecting CEBM patterns and rebound mechanisms and building upon generalized, empirically-based databases of both domains. It can also inform the identification of monitoring indicators (Leipold et al., 2023; Lowe et al., 2024). For instance, moral hazard in car-sharing could be monitored by tracking damage reports per use against historical averages to identify irresponsible users of shared assets.

However, to apply the semantic network effectively, it is crucial to understand its purpose as a tool for proactive early-stage reflection on rebound pathways. Although the study employs quantitative measures, such as the number of connections, it cannot yet assess the relevance of rebound mechanisms in specific cases (**Limitation 1**). A node's prominence indicates theoretically relevant associations, not its actual magnitude or likelihood. A single, less-connected pathway could still have a disproportionately large impact in a specific context. Furthermore, the focus on generalized archetypes blurs the nuances of specific cases (**Limitation 2**) as a single trigger may mask different underlying dynamics. For example, 'decreased cost' for a renter and 'new revenues' for a lender both influence a user's budget but can trigger behaviors that will unfold differently over time. The purpose of this semantic network, therefore, is to reveal the scope of potential systemic responses as the causal complexity of RE is contained within each mechanism. For example, it can inform investigations into the temporal dynamics and contextual dependencies that the map itself does not capture, for which simulation-based approaches are ideal (Guzzo et al., 2023; Koide et al., 2023; Metic et al., 2024).

### Biases in describing CEBMs and rebound mechanisms limit RE identification potential

The analysis in the research reveals that the current state of knowledge on CE-rebound, as represented by its two state-of-the-art catalogs (Guzzo et al., 2024; Pieroni et al., 2021), is biased and incomplete (**Limitation 3**). While the financial/economic factors dominate, critical factors are marginalized: links to consumer choices and physical constraints were not only a minority but were based on weaker, non-explicit evidence (Table C-5, SI3). For example, consumer choices (CT3) were somewhat explicit (~ 37%), but a majority were extrapolated from other contexts or similar patterns (~ 45%). Also, several triggers and drivers in the investigation of CEBM patterns had no corresponding mechanism in the catalog, notably, space-saving (T2.3), moral hazard (T3.3 and T5.2), and moral credits (T3.4 and T5.3). While research acknowledges the effect of space savings (e.g., Azevedo, 2014), and moral-related effects leading to RE (e.g., Bączyk et al., 2024), they had not yet been formally integrated into catalogs employed in this study. In addition, no trigger stimulation was associated with socio-cultural influences (CT4), while they have been linked to RE (e.g., Azevedo, 2014). This omission likely reflects a two-sided conceptual challenge: the generalized, abstract nature of CEBM patterns may inherently filter out such contextual factors. At the same time, the associated mechanisms may remain conceptually unclear in the literature and thus difficult to systematically identify.

These blind spots stem from a deeply ingrained dependence on a value-capture paradigm to understand CEBMs (Richardson, 2008; Schultz et al., 2024) and on neoclassical economics to explain RE (Andrew & Pigosso, 2024). Ultimately, this leads to a form of *rebound myopia*, characterized by a limited capacity to communicate about RE beyond a narrow set of economic factors. However, the non-identification of a link in the source catalogs does not imply its absence in the real world – other factors, patterns, or mechanisms may turn relevant as the semantic network is expanded. Addressing these limitations requires a dual approach: expanding the CEBM pattern language and systematically reviewing the current rebound mechanism catalogs to include this broader range of triggers and drivers, which is happening in the field (Andrew et al., 2026; see van der Loo & Pigosso, 2024). Our framework (i.e., ontology, semantic network, and associated procedures) serves as the essential bridge for monitoring the association between the two knowledge domains as they evolve.

### The research expands the scope of understanding RE in sustainability, but it is still limited

The research sheds light on the RE merging from effectiveness interventions, a category identified as an open field in the RE literature that has historically prioritized efficiency (Guzzo, Andrew, et al., 2024; Sorrell et al., 2020). The analysis demonstrates that all three strategic approaches (i.e., efficiency, effectiveness and sufficiency) are similarly prone to RE. In addition, we were able to pinpoint a systematic association between specific CEBM patterns and rebound mechanisms, challenging the proposition that RE “are not necessarily tied to specific CE strategies” (Siderius & Poldner, 2021, p. 8).

However, the scope of this research is limited to a specific type of sustainability-oriented action and overlooks secondary benefits (**Limitation 4**). Although this conceptual lens was developed using CEBM patterns, it can be adapted to examine RE in other sustainability-focused interventions, such as product design, socio-technical interventions, and policy tools. We suggest that our framework may serve as a useful link between these types of interventions and RE, a hypothesis warranting further research. In addition, these lenses can be inverted: they can be used to stimulate secondary benefits, which share similar causal structures (Bączyk et al., 2024; Guzzo & Pigosso, 2024). The quest to understand the RE phenomenon should therefore be pursued not only to prevent RE but also to reinforce positive impacts, thereby securing the full sustainability gains of the interventions.

### A foundational framework for advancing rebound research in CE and beyond

A fundamental contribution of this research is the operationalization of a causal ontology that helps the research of RE in CEBM evolve from categorizing isolated cases to identifying patterns based on relational knowledge, the foundation of higher cognition and applicability (Halford et al., 2010). Our work enhances current analytical frameworks for exploring RE in CE (Castro et al., 2022; Chen, 2021; Ferrante, 2026; Ferrante et al., 2024; Metic & Pigosso, 2022) by offering a structure that systematically associates CEBM patterns and rebound mechanisms through textual analysis and network synthesis and visualization.

These theoretical advancements are grounded in a robust and replicable methodological framework, supported by a set of validation principles. Content validity was established by grounding the ontology and the analysis in the state-of-the-science (Guzzo, Walrave, et al., 2024; Pieroni et al., 2021), representing the frontier at both knowledge domains. Process validity and replicability (Krippendorff, 2004) were ensured through a rigorous coding protocol that included interpretive and analogical evidence. This methodological choice was essential for broadening the analysis beyond economic determinants of RE and extending its applicability to the B2C context. We address the inherent subjectivity of this approach by using a transparent evidence system for each coded link and by providing access to the datasets and scripts for network visualizations, enabling validation by the research community.

The combination of visualization techniques contributes to process validity in some ways. First, the Sankey diagrams enforce the ontology's sequential causal logic, from CEBM patterns to rebound mechanisms. The chord diagrams show the overall structural interconnections, providing a view of all types of relationships simultaneously. The color system further exposes triggers and drivers as hubs that connect CEBM patterns and rebound mechanisms dynamics. The representations also make explicit two types of connections and help identify the most and least interconnected elements. The use case reinforces the framework's pragmatic validity, which has also been demonstrated in a preliminary case study of the semantic network elsewhere (Guzzo & Pigosso, 2025).

The value for research is demonstrated through the structural insights enabled by the semantic network. It shed light on the limits of the 'win–win' narrative in delivering sustainability (Calisto Friant et al., 2020; Siderius & Poldner, 2021), making explicit how known CEBM patterns can activate several rebound mechanisms. By systematically connecting constructs often studied in isolation using an explicit ontology, this work enhances our ability to understand why specific CE strategies may fail to deliver their sustainability potential and provides the foundation for more holistic assessments that can potentialize those benefits (Calisto Friant et al., 2020; Font-Vivanco et al., 2022).

Finally, the contributions of this research are the outcomes of employing semantic network analysis with the following characteristics: i. follows a causal ontology, ii. employs an interpretation-based approach, iii. considers state-of-the-art knowledge sets as the source materials, iv. employs qualitative content analysis procedures appropriate for those sets, v. consolidates the knowledge using network synthesis and visualization procedures and relational matrices. However, the results are still reliant on the use of well-structured catalogs of text and the analysts’ interpretation of the association of those texts with the ontology (**limitation 5**).

While the use of well-structured knowledge sets was useful for validating the framework (i.e., ontology, existing network, and associated procedures), it is possible and encouraged to employ it with less structured knowledge sets. In that direction, a significant next step involves the formalization of a thesaurus to manage synonyms and conceptual overlaps for the ontology’s elements and relationships, towards decreasing the interpretation required for the analysis of less structured content. There is also space to further scrutinize the ontology by, for example, clarifying the role of drivers in the propagation of change. The semantic network can be elaborated through triangulation, which involves systematic literature reviews to explore the identified biases and case studies to test the associations with real-world dynamics. As additional evidence is collected, network-analytic procedures can help identify unforeseen patterns. Ultimately, this validated framework provides a solid foundation for future explanatory research, as its core logic aligns with dynamic modelling and simulation tools that can provide further insight for effective learning and governance of RE in CEBM and beyond.

## Conclusion

There is an urgent need to clarify the systematic occurrence of RE in CE. This research explored the associations between CEBM patterns and rebound mechanisms by employing semantic network analysis, grounded in a causal ontology, applied to knowledge sets of CEBM patterns and archetypal rebound mechanisms. The ontology, the semantic network and complementary procedures contribute to research and practice. The study provides three main contributions: (1) a first semantic network of the associations between CEBM patterns and rebound mechanisms, offering important structural insights; (2) a clear positioning of triggers and drivers as the essential, actionable links between CEBM and RE; and (3) an operational causal ontology and complementary analytical procedures to support RE analysis within sustainability transitions. There is also considerable potential for further research, indicated by the identified limitations.

Overall, this research supports the progress of the RE field from merely classifying individual cases to exploring underlying relationships. It provides a systematic, knowledge-based foundation for understanding why strategies such as CEBM may not fully achieve their sustainability objectives. The semantic network and complementary analytical processes provide a structured basis for future empirical research, which remains essential for further validating these potential pathways. Ultimately, it equips decision-makers with an analytical framework to move from reactive responses to proactive reflection and resolutions. By carefully analyzing the causes and factors behind rebound effects, it becomes possible to design reboundless interventions.

## Summaries

### Supplementary Information SI1:

This Supplementary Information provides the material used in the analysis and an illustrative application of the analytical framework, as indicated in Research Stages 2 and 3 in the Methodology (Section 3).

### Supplementary Information SI2:

This supplementary information provides the complete analytical taxonomies with definitions and examples, complementing the results presented in Section 4.

### Supplementary Information SI3:

This supplementary information provides the cross-tabulation analyses that support the research insights presented in the results (Section 4).

## Supplementary Information

Below is the link to the electronic supplementary material.

Supplementary file1 (PDF 380 KB)—Supplementary Information A: This Supplementary Information provides the material used in the analysis and an illustrative application of the analytical framework, as indicated in Research Stages 2 and 3 in the Methodology (Section 3).

Supplementary file2 (PDF 252 KB)—Supplementary Information B: This supplementary information provides the complete analytical taxonomies with definitions and examples, complementing the results presented in Section 4.

Supplementary file3 (PDF 520 KB)—Supplementary Information C: This supplementary information provides the cross-tabulation analyses that support the research insights presented in the results (Section 4).

## Data Availability

The full database of the relationships between CEBM patterns, actors, triggers and drivers, and rebound mechanisms is available in (https://doi.org/10.11583/DTU.29528174).
