# Supplementary material for: Bridging circular economy business models and rebound effects: Mapping systemic associations with semantic network analysis
Source: J Ind Ecol. 2026 Jul 15;30(4):1629–43. doi: 10.1007/s44498-026-00110-3 (PMC13451322; doi:10.1007/s44498-026-00110-3)
Supplement: Supplementary file 1 [file 44498_2026_110_MOESM1_ESM.pdf]

SUPPLEMENTARY INFORMATION 1 FOR:

Guzzo, D. & Pigosso, D. Bridging circular economy business models and rebound effects: Mapping systemic associations with semantic network analysis. *Journal of Industrial Ecology.*

Summary

This Supplementary Information provides the material used in the analysis and an illustrative application of the analytical framework, as indicated in Research Stages 2 and 3 in the Methodology (Section 3).

Table A-1 - Relationship between CEBM patterns (P) and pattern cards (C) identified

#	P01	P02	P03	P04	P05	P06	P07	P08	P09	P10	P11	P12	P13	P14	P15	P16	P17	P18	P19	P20	P21	P22	P23	P24	--
C01	x																								
C02	x																								
C03	x																								
C04			x																						
C05		x																							
C06				x																					
C07					x																				
C08				x																					
C09				x																					
C10						x																			
C11						x																			
C12							x																		
C13							x																		
C14								x																	
C15							x																		
C16							x																		
C17									x																
C18										x															
C19											x														
C20											x														
C21												x													
C22													x												
C23														x											
C24														x											
C25																							x		
C26															x										
C27															x										
C28															x										
C29																x									
C30																	x								
C31																									x
C32																		x							
C33																		x							
C34																			x						
C35																				x					
C36																					x				
C37																						x			
C38																								x	
C39																								x	
C40																								x	
C41																								x	
C42																								x	
C43																								x	
C44																								x	

Table A-2 - Description of a CEBM pattern (PB06: Collaborative services) used as input to Research Stage 2

**Pattern**	**What usually is the problem? Structural waste origin**	**How to address it with a new business model configuration? Solution description**	**To think about... How could you adopt this business model pattern?**	**Examples of companies**	**Potential effects on resource consumption reduction**
P06: Collaborative services	- Customers have temporary needs for using products due to their dynamic lifestyle , needs for frequent changes in aesthetics, or size/ dimensions/ volume.<br>- As a consequence, products are used for shorter periods than their real life-time, generating unnecessary costs (and sometimes missed opportunities to trade it further) for the users.	- This offering is based on solutions that enable sequential users to access or use products temporally.<br>- Usually, digital and physical platforms are established as key channels and coordinated by the manufacturer or controlled by an integrator with expertise in logistics and digital technologies (e.g. marketplaces).<br>- Transactions can occur in a peer-to-peer or peer-to-integrator format.<br>- Different transactional arrangements are possible (e.g. pay-per-use or subscription, listing fees, commissions per transactions).	How could you facilitate access to your products and attract new customers by creating a sharing system that enables sequential use of products by several users with the potential to reduce idleness of products and enable better use and for longer?	• Zentec Pool System - pallets in time<br>• Drive Now - car sharing<br>• ShareDesk - Online marketplace for sharing/pooling workspaces and offices<br>• FLOOW2 - digital marketplace for equipment and services sharing (within regions)<br>• Equipment Share - digital marketplace for machinery and equipment rental with smart asset management solution	Displace more resource intensive systems and intensified product usage.

Table A-3 – Description of pattern cards (C10: Product as collaborative service: sharing and C11: Product as collaborative service: pooling platforms complementary to PB06: Collaborative services) used as input to Research Stage 2

**Pattern card**	**Description/Question**	**Case**
C10: Product as collaborative service: sharing platforms based on transaction fee or subscription	How could you facilitate access to products and attract new customers by creating a sharing platform that enables sequential use of products by several users with the potential to reduce idleness of products?	Lena fashion library - Sharing and borrowing of clothes
C11: Products as collaborative service: pooling platforms	How could you facilitate access to products and attract new customers by creating a pooling platform that enables simultaneous use of products by several users with the potential to reduce idleness of products and enable better use and for longer?	ShareDesk - Pooling of workspaces and meeting rooms

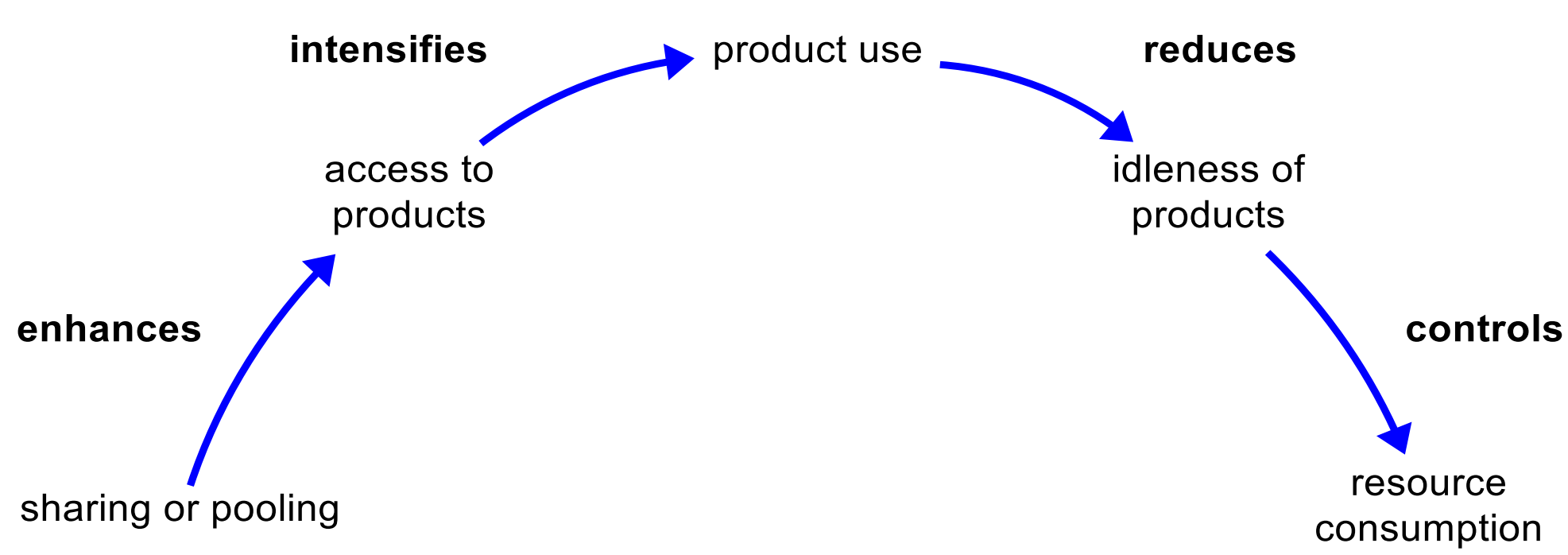

Figure A-1 – Visual representation of the theory of intended effects of CEBM pattern PB06: Collaborative services, where enhanced access to products through sharing/pooling intensifies product use and reduces the idleness of products, thereby controlling resource consumption.

Table A-4 – Stimulation instances of triggers and drivers of the CEBM pattern PB06: Collaborative services

Actor	Change in quantity, quality or state	Trigger/Driver	Cause of change	Known Trigger/Driver	Evidence
A1.1: Manufacturer/ Service provider	New	Revenues	from the platform use (e.g., commission fees)	TD1.1: Revenues	Explicit
A1.2: Company customer (purchaser/renter)	Decreased	costs	from fleet acquisition	TD1.2: Operational costs	Extrapolated
	Less	responsibility	due to no ownership model	TD5.2: Moral hazard	Extrapolated
A1.3: Company seller/Lender	New	revenues	from owned products in the platform	TD1.1: Revenues	Extrapolated
A2.1: Individual purchaser/renter	Decreased	costs to access or own a product	from access to products through collaborative services	TD1.3: Budget	Explicit
	Increased	flexibility to solve temporary needs for products	from access to products through collaborative services	TD3.2: Convenience/ Perception	Interpretive
	Less	responsibility	due to no ownership model	TD3.3: Moral hazard	Extrapolated
A2.2: Individual seller/Lender	New	revenues	from owned products in the platform	TD1.3: Budget	Explicit
CA3. Good	—	Substitutability of the shared product to other options	—	TD6.1: Substitutability of good	Explicit
	—	Time-intensity of the shared product to other options	—	TD6.3: Time-intensity to consume	Extrapolated

Table A-5 – Triggers and drivers for the RMA1.4: Re-spending mechanism for one actor

Actor	Triggers	Driver
Individual consumer	Cost of consumption, Budget available	N/A
Good	N/A	Substitutability, Resource intensity
