# Supplementary material for: Bridging circular economy business models and rebound effects: Mapping systemic associations with semantic network analysis
Source: J Ind Ecol. 2026 Jul 15;30(4):1629–43. doi: 10.1007/s44498-026-00110-3 (PMC13451322; doi:10.1007/s44498-026-00110-3)
Supplement: Supplementary file 2 [file 44498_2026_110_MOESM2_ESM.pdf]

SUPPLEMENTARY INFORMATION 2 FOR:

Guzzo, D. & Pigosso, D. Bridging circular economy business models and rebound effects: Mapping systemic associations with semantic network analysis. *Journal of Industrial Ecology.*

Summary

This supplementary information provides the complete analytical taxonomies with definitions and examples, complementing the results presented in Section 4.

Table B-1 – CEBM patterns categorized into efficiency ('effi.'), effectiveness ('effe.'), sufficiency ('suff.') and enabling

**CEBM Pattern**	**Description**	**Type of CEBM pattern**			
		**Effi. (PA)**	**Effe. (PB)**	**Suff. (PC)**	**Enabling (PD)**
P01: Data-driven services	Enhanced real-time information about products, enabling tracking usage, location, and failure prediction				x (P04; P05; P06; P07; P08; P10; P11; P12; P13; P14; P16; P17; P23)
P02: Dematerialized services	Provision of digitalized products leading to dematerialization (i.e., less resource-intensive products) and decreased resources or energy consumption	x			
P03: Consumption rationalization	Enhanced information on consumption intensity, leading to increased awareness and decreased consumption of resources or energy			x	
P04: Result-as-a-service	Enhanced access to results/performance/experience/ functionality, reducing the reliance on products and minimizing the use of resources or energy		x		
P05: Activity management agreement	Enhanced access to agreed activity, reducing the reliance on products and minimizing the use of resources or energy		x		
P06: Collaborative services	Enhanced access to products through sharing/pooling, reducing the idleness of products		x		
P07: Product as a service	Enhanced access to products through pay-per-period/pay-per-use/long-term/short-term contracts, reducing the idleness of products		x		
P08: Product as a service (in pre-configured packages)	Enhanced access to products through subscription contracts, reducing the idleness of products		x		
P09: Lifetime products	Enhanced durability of products through product design, increasing product longevity		x		
P10: Life care services	Enhanced durability of products through life extension services, increasing product longevity		x		
P11: Buy-back	Enhanced conditions and customers' motivations to return products, improving the collection of good-condition products				x (P13)
P12: New life cycles (services)	Additional life cycle for products through services, increasing product longevity		x		
P13: New life cycles (products)	Additional life cycle for products through reselling, increasing product longevity		x		
P14: Trade platforms (durables)	Enhanced access to reuse durable products or trade surplus of consumables, increasing the use/consumption of products/consumables in good conditions		x		
P15: Circular supplies	Increased use of circular supplies (e.g., biodegradable, renewable, recovered, and recycled), decreasing the need for virgin resources	x			
P16: Industrial symbiosis	Increased exchange of waste and by-products, leading to decreased waste generation and virgin resources need	x			
P17: Assets as service	Enhanced access to internal assets, leading to reduced idleness of products		x		
P18: On-demand or local manufacturing	Enhanced production (e.g., on-demand or local), leading to less need for stocks and unnecessary transportation	x			
P19: Life extension activities	Enhanced capabilities and support network for life extension services, increasing the longevity of assets				x (P10; P12)
P20: Own reuse/refurb	Enhanced internal capabilities for operationalization of reuse or refurbishment				x (P13)
P21: Own reman	Enhanced internal capabilities for the operationalization of remanufacturing				x (P13)
P22: Own recycling	Enhanced internal capabilities for the operationalization of recycling				x (P15)
P23: Bio-based products	Enhanced reprocessing of bio-based materials, leading to decreased generation of waste and less need for virgin resource consumption	x			
P24: Collaborate	Enhanced collaboration for the operationalization of more extended use of products, life extension services, digital services, financial services, infrastructure for collection, reprocessing, recycling, and end-of-life treatment				x (All)

Table B-2 – Actors identified performing CEBM patterns   Triggers and drivers stimulated by CEBM patterns

Type of actor	Actor	Role
A1. Company	A1.1: Manufacturer/Service provider	Value proposition provider
	A1.2: Customer (purchaser/renter)	Company customers purchasing or renting the solution
	A1.3: Seller/Lender	Companies selling or lending in platform-based solutions
	A1.4: Supplier	Suppliers of components and materials
A2. Individual	A2.1: Customer (purchaser/renter)	Individual customers purchasing or renting the solution
	A2.2: Seller/Lender	Individuals selling or lending in platform-based solutions
A3. Good	A3.1: Machine/Product/Service	Goods that represent the value proposition
	A3.2: Production process	Processes enabling the production of goods

Table B-3 – Triggers and drivers stimulated by CEBM patterns

Type of trigger or driver	Trigger or driver	Exemplary instances (incl. related patterns)
TD1. Economic/ Financial	TD1.1: Revenues	New revenues from the contract (P04, P05, P07, P08, P10)
	TD1.2: Operational costs	Decreased acquisition costs from replacing products less frequently (P09, P10)
	TD1.3: Budget	Decreased costs to own a product by using additional life cycles of the same product (P12)
	TD1.4: Excess of supply of product	Decreased individual consumption from enhanced rationality of consumption (P03)
	TD1.5: Excess of supply of process	Decreased company consumption from enhanced rationality of consumption (P03)
TD2. Physical constraint	TD2.1: Production time	Additional time for production from the enhanced ability to track usage and location (P01)
	TD2.2: Consumption time	Additional time for consumption from the enhanced ability to track usage and location (P01)
	TD2.3: Space savings	Increased space savings from decreased need for material stocks (P02)
TD3. Consumer choice	TD3.1: Awareness of consumption intensity	Increased awareness of consumption intensity (P03)
	TD3.2: Convenience/Perception	Increased flexibility to solve temporary needs for products (P04, P06, P07, P08)
	TD3.3: Moral hazard	Less responsibility due to no ownership model (P04, P06, P07, P08)
	TD3.4: Moral credits	Increased moral credits from non-monetary incentives (P11)
TD4. Socio-cultural influences	-	-
TD5. Company choice	TD5.1: Reinvestment in innovation	New opportunities for innovation from reprocessed materials (P23)
	TD5.2: Moral hazard	Less responsibility due to no ownership model (P04, P05, P06, P07, P08, P17)
	TD5.3: Moral credits	Increased moral credits from non-monetary incentives (P11)
TD6. Goods' attributes	TD6.1: Substitutability of good	Substitutability of the asset to other options (P17)
	TD6.2: Substitutability of process	Substitutability of the circular material to virgin material (P15)
	TD6.3: Time-intensity to consume	Time-intensity of the shared product to other options (P06)
	TD6.4: Resource intensity of good	Resource intensity of the longer-lifetime product to other options (P09, P10)
	TD6.5: Resource intensity of process	Relative costs of local production to mass production and worldwide shipping (P18)

Table B-4 – Classification of the rebound mechanisms considering the actors involved

Rebound Mechanism	Type of rebound mechanism			
	**RMA. Consumption**	**RMB. Production – Single company**	**RMC. Production – Multiple companies**	**RMD. Consumption and production**
RM1.1: Income	x			
RM1.2: Consumption time	x			
RM1.3: Motivational consumption	x			
RM1.4: Re-spending	x			
RM2.1: Output		x		
RM2.2: Production time		x		
RM2.3: Re-investment		x		
RM2.4: Cost-dependent output			x	
RM3.1: Substitution	x			
RM3.2: Motivational substitution	x			
RM3.3: Factor substitution		x		
RM3.4: Composition substitution			x	
RM3.5: Sectoral allocation			x	
RM4.1: Demand adjustment				x
RM4.3: Demand adjustment with investment adjustment				x
RM4.4: Re-design		x		
RM4.5: Supply adjustment			x	
RM4.6: Producer demand adjustment			x	
RM4.7: Sector demand adjustment			x	
RM5.1: Economies of scale				x
RM5.2: Market price				x
RM5.3: Labor income				x
RM5.5: New economic activity				x

Table B-5 – Constituent elements of the semantic network organized into classes of elements

**CEBM Patterns (P)**			
PA. Efficiency	PB. Effectiveness	PC. Sufficiency	PD. Enabling
PA02: Dematerialized services<br>PA15: Circular supplies<br>PA16: Industrial symbiosis<br>PA18: On-demand or local manufacturing<br>PA23: Bio-based products	PB04: Result-as-a-service<br>PB05: Activity management agreement<br>PB06: Collaborative services<br>PB07: Product as a service<br>PB08: Product as a service (in pre-configured packages)<br>PB09: Lifetime products<br>PB10: Life care services<br>PB12: New life cycles (services)<br>PB13: New life cycles (products)<br>PB14: Trade platforms (durables)<br>PB17: Assets as service	PC03: Consumption rationalization	PD01: Data-driven services<br>PD11: Buy-back<br>PD19: Life extension activities<br>PD20: Own reuse or refurbishment<br>PD21: Own remanufacturing<br>PD22: Own recycling<br>PD24: Collaborate

**Actors (A)**		
A1. Company	A2. Individual	A3. Good
A1.1: Manufacturer/Service provider<br>A1.2: Customer (purchaser/renter)<br>A1.3: Seller/Lender<br>A1.4: Supplier	A2.1: Customer (purchaser/renter)<br>A2.2: Seller/Lender	A3.1: Machine/Product/Service<br>A3.2: Production process

**Triggers and Drivers (TD)**					
TD1. Economic/ Financial	TD2. Physical constraint	TD3. Consumer choice	TD4. Socio-cultural influences	TD5. Company choice	TD6. Goods' attributes
TD1.1: Revenues<br>TD1.2: Operational costs<br>TD1.3: Budget<br>TD1.4: Excess of supply of product<br>TD1.5: Excess of supply of process	TD2.1: Production time<br>TD2.2: Consumption time<br>TD2.3: Space savings	TD3.1: Awareness of consumption intensity<br>TD3.2: Convenience /Perception<br>TD3.3: Moral hazard<br>TD3.4: Moral credits		TD5.1: Re-investment in innovation<br>TD5.2: Moral hazard<br>TD5.3: Moral credits	TD6.1: Substitutability of good<br>TD6.2: Substitutability of process<br>TD6.3: Time-intensity to consume<br>TD6.4: Resource intensity of good<br>TD6.5: Resource intensity of process

**Rebound Mechanisms (RM)**			
RMA. Consumption	RMB. Production – Single company	RMC. Production – Multiple companies	RMD. Consumption and production
RMA1.1: Income<br>RMA1.2: Consumption time<br>RMA1.3: Motivational consumption<br>RMA1.4: Re-spending<br>RMA3.1: Substitution<br>RMA3.2: Motivational substitution	RMB2.1: Output<br>RMB2.2: Production time<br>RMB2.3: Re-investment<br>RMB3.3: Factor substitution<br>RMB4.4: Re-design	RMC2.4: Cost-dependent output<br>RMC3.4: Composition substitution<br>RMC3.5: Sectoral allocation<br>RMC4.5: Supply adjustment<br>RMC4.6: Producer demand adjustment<br>RMC4.7: Sector demand adjustment	RMD4.1: Demand adjustment<br>RMD4.3: Demand adjustment with investment adjustment<br>RMD5.1: Economies of scale<br>RMD5.2: Market price<br>RMD5.3: Labor income<br>RMD5.5: New economic activity
