# Supplementary material for: Bridging circular economy business models and rebound effects: Mapping systemic associations with semantic network analysis
Source: J Ind Ecol. 2026 Jul 15;30(4):1629–43. doi: 10.1007/s44498-026-00110-3 (PMC13451322; doi:10.1007/s44498-026-00110-3)
Supplement: Supplementary file 3 [file 44498_2026_110_MOESM3_ESM.pdf]

SUPPLEMENTARY INFORMATION 3 FOR:

Guzzo, D. & Pigosso, D. Bridging circular economy business models and rebound effects: Mapping systemic associations with semantic network analysis. *Journal of Industrial Ecology.*

Summary

This supplementary information provides the cross-tabulation analyses that support the research insights presented in the results (Section 4).

Table C-1 - Actors (A) performing CEBM patterns (P) and stimulating triggers and drivers (TD). A1.1: Manufacturer/Service provider; A1.2: Customer (purchaser/renter); A1.3: Seller/Lender; A1.4: Supplier; A2.1: Customer (purchaser/renter); A2.2: Seller/Lender; A3.1: Machine/Product/Service; A3.2: Production process.

	**A1. Company**					**A2. Individual**			**A3. Good**			**Total**	**Count Actors**
**Patterns (P) \ Actor (A)**	**A1.1**	**A1.2**	**A1.3**	**A1.4**	**CA1**	**A2.1**	**A2.2**	**CA2**	**A3.1**	**A3.2**	**CA3**		
PD01: Data-driven services	2	2			4	1		1			-	5	3
PA02: Dematerialized services	3	3			6	2		2			-	8	3
PC03: Consumption rationalization	1	2			3	3		3			-	6	3
PB04: Result-as-a-service	2	3			5	3		3	1		1	9	4
PB05: Activity management agreement	2	3			5			-	1		1	6	3
PB06: Collaborative services	1	2	1		4	3	1	4	2		2	10	6
PB07: Product as a service	1	3			4	3		3	2		2	9	4
PB08: Product as a service (in pre-configured packages)	1	3			4	3		3	2		2	9	4
PB09: Lifetime products	1	1			2	1		1	2		2	5	4
PB10: Life care services	1	2			3	1		1	2		2	6	4
PD11: Buy-back	2	2			4	2		2			-	6	3
PB12: New life cycles (services)	1	1			2	1		1			-	3	3
PB13: New life cycles (products)	1	1			2	1		1			-	3	3
PB14: Trade platforms (durables)	1	1			2	1	2	3	1		1	6	5
PA15: Circular supplies	1			1	2	1		1	1		1	4	4
PA16: Industrial symbiosis		1		1	2			-	1		1	3	3
PB17: Assets as service	5				5			-	1		1	6	2
PA18: On-demand or local manufacturing	4			1	5			-		1	1	6	3
PD19: Life extension activities	1	1			2			-			-	2	2
PD20: Own reuse/refurb					-			-			-	-	-
PD21: Own reman					-			-			-	-	-
PD22: Own recycling					-			-			-	-	-
PA23: Bio-based products	2			2	4			-	1		1	5	3
PD24: Collaborate					-			-			-	-	-
Total	33	31	1	5	70	26	3	29	17	1	18	117	

Table C-2 – Triggers and drivers (TD) stimulated by CEBM pattern (P). TD1.1: Revenues; TD1.2: Operational costs; TD1.3: Budget; TD1.4: Excess of supply of product; TD1.5: Excess of supply of process TD2.1: Production time; TD2.2: Consumption time; TD2.3: Space savings; TD3.1: Awareness of consumption intensity; TD3.2: Convenience/Perception; TD3.3: Moral hazard; TD3.4: Moral credits; TD5.1: Reinvestment in innovation; TD5.2: Moral hazard; TD5.3: Moral credits; TD6.1: Substitutability of good; TD6.2: Substitutability of process; TD6.3: Time intensity to consume; TD6.4: Resource intensity of good; TD6.5: Resource intensity of process.

	TD1. Economic/Financial						TD2. Physical constraints				TD3. Consumer choices					TD5. Company choices				TD6. Goods and services` attributes						Total	Count TD
**Pattern (P) \ Trigger or driver (TD)**	**1.1**	**1.2**	**1.3**	**1.4**	**1.5**	**TD1**	**2.1**	**2.2**	**2.3**	**TD2**	**3.1**	**3.2**	**3.3**	**3.4**	**TD3**	**5.1**	**5.2**	**5.3**	**TD5**	**6.1**	**6.2**	**6.3**	**6.4**	**6.5**	**TD6**		
PD01: Data-driven services	1	2				3	1	1		2																5	4
PA02: Dematerialized services		2	1			3			3	3						2			2							8	4
PC03: Consumption rationalization	1	1	1	1	1	5				-	1				1											6	6
PB04: Result-as-a-service	1	3	1			5				-		1	1		2		1		1	1					1	9	7
PB05: Activity management agreement	1	3				4				-							1		1	1					1	6	4
PB06: Collaborative services	2	1	2			5				-		1	1		2		1		1	1		1			2	10	8
PB07: Product as a service	1	2	1			4				-		1	1		2		1		1	1			1		2	9	8
PB08: Product as a service (in pre-configured packages)	1	2	1			4				-		1	1		2		1		1	1			1		2	9	8
PB09: Lifetime products	1	1	1			3				-										1			1		2	5	5
PB10: Life care services	2	1	1			4				-										1			1		2	6	5
PD11: Buy-back	1	2	1			4				-				1	1			1	1							6	5
PB12: New life cycles (services)	1	1	1			3				-																3	3
PB13: New life cycles (products)	1	1	1			3				-																3	3
PB14: Trade platforms (durables)	1	1	3			5				-										1					1	6	4
PA15: Circular supplies	1	1				2				-		1			1						1				1	4	4
PA16: Industrial symbiosis	1	1				2				-											1				1	3	3
PB17: Assets as service	1	1				2	1		1	2							1		1	1					1	6	6
PA18: On-demand or local manufacturing	2	2				4			1	1														1	1	6	4
PD19: Life extension activities	1	1				2				-																2	2
PD20: Own reuse/refurb						-				-																	0
PD21: Own reman						-				-																	0
PD22: Own recycling						-				-																	0
PA23: Bio-based products	2	1				3				-						1			1		1				1	5	4
PD24: Collaborate						-				-																	0
Total	23	30	15	1	1	70	2	1	5	8	1	5	4	1	11	3	6	1	10	9	3	1	4	1	18	117	

Table C-3 - Types of triggers and drivers (TD) stimulated by types of CEBM pattern (P). TD1. Economic/Financial, TD2. Physical constraints, TD3. Consumer choices, TD5. Company choices, TD6. Goods' attributes

Type of patterns (P) \ Types of triggers and drivers (TD)	Total CEBM patterns	TD1.	TD2.	TD3.	TD4.	TD5.	TD6.	Total	Average per pattern
PA. Efficiency	5	14	4	1	-	3	4	26	5.2
PB. Effectiveness	11	42	2	8	-	6	14	72	6.54
PC. Sufficiency	1	5	-	1	-	-	-	6	6
PD. Support	7	9	2	1	-	1	-	11	1.57
Total	24	70	8	11	0	10	18	117	4.88

Table C-4 - Types of triggers and drivers (TD) stimulated by actors (A) performing CEBM patterns (P). A1.1: Manufacturer/Service provider; A1.2: Customer (purchaser/renter); A1.3: Seller/Lender; A1.4: Supplier; A2.1: Customer (purchaser/renter); A2.2: Seller/Lender; A3.1: Machine/Product/Service; A3.2: Production process.

	A1. Company					A2. Individual			A3. Good			
**Trigger or driver (TD) \ Actor (A)**	**A1.1**	**A1.2**	**A1.3**	**A1.4**	**A1**	**A2.1**	**A2.2**	**A2**	**A3.1**	**A3.2**	**A3**	**Total**
TD1.1: Revenues	17	1	1	4	23							23
TD1.2: Operational costs	9	20		1	30							30
TD1.3: Budget						12	3	15				15
TD1.4: Excess of supply of product						1		1				1
TD1.5: Excess of supply of process		1			1							1
TD1. Economic/Financial	26	22	1	5	54	13	3	16				70
TD2.1: Production time	1	1			2							2
TD2.2: Consumption time						1		1				1
TD2.3: Space savings	3	1			4	1		1				5
TD2. Physical constraints	4	2			6	2		2				8
TD3.1: Awareness of consumption intensity						1		1				1
TD3.2: Convenience/Perception						5		5				5
TD3.3: Moral hazard						4		4				4
TD3.4: Moral credits						1		1				1
TD3. Consumer choices						11		11				11
TD5.1: Reinvestment in innovation	2	1			3							3
TD5.2: Moral hazard	1	5			6							6
TD5.3: Moral credits		1			1							1
TD5. Company choices	3	7			10							10
TD6.1: Substitutability of good									9		9	9
TD6.2: Substitutability of process									3		3	3
TD6.3: Time intensity to consume									1		1	1
TD6.4: Resource intensity of good									4		4	4
TD6.5: Resource intensity of process										1	1	1
TD6. Goods' attributes									17	1	18	18
Total	33	31	1	5	70	26	3	29	17	1	18	117

Table C-5 - Level of evidence of triggers and drivers (TD) stimulation per type

	**i. Explicit**		**ii. Interpretive**		**iii. Extrapolated**		**Total**
**Trigger or driver (TD) \ Level of evidence**	**Count**	**%**	**Count**	**%**	**Count**	**%**	**Count**
TD1.1: Revenues	12	52.2%	10	43.5%	1	4.3%	23
TD1.2: Operational costs	17	56.7%	5	16. 7%	8	26.7%	30
TD1.3: Budget	6	40.0%	6	40.0%	3	20.0%	15
TD1.5: Excess of supply of process	1	100.0%		0.0%		0.0%	1
TD1.4: Excess of supply of product	1	100.0%		0.0%		0.0%	1
TD1. Economic/Financial	37	52.9%	21	30.0%	12	17.1%	70
TD2.1: Production time		0.0%	2	100.0%		0.0%	2
TD2.2: Consumption time		0.0%		0.0%	1	100.0%	1
TD2.3: Space savings	1	20.0%	1	20.0%	3	60.0%	5
TD2. Physical constraints	1	12.5%	3	37.5%	4	50.0%	8
TD3.1: Awareness of consumption intensity	1	100.0%		0.0%		0.0%	1
TD3.2: Convenience/Perception	3	60.0%	1	20.0%	1	20.0%	5
TD3.3: Moral hazard		0.0%		0.0%	4	100.0%	4
TD3.4: Moral credits		0.0%	1	100.0%		0.0%	1
TD3. Consumer choices	4	36.4%	2	18.2%	5	45.4%	11
TD5.1: Reinvestment in innovation	2	66. 7%	1	33.3%		0.0%	3
TD5.2: Moral hazard		0.0%	2	33.3%	4	66.7%	6
TD5.3: Moral credits		0.0%	1	100.0%		0.0%	1
TD5. Company choices	2	20.0%	4	40.0%	4	40.0%	10
TD6.1: Substitutability of good	1	11.1%	8	88.9%		0.0%	9
TD6.2: Substitutability of process		0.0%	3	100.0%		0.0%	3
TD6.3: Time intensity to consume		0.0%	1	100.0%		0.0%	1
TD6.4: Resource intensity of good		0.0%	4	100.0%		0.0%	4
TD6.5: Resource intensity of process		0.0%	1	100.0%		0.0%	1
TD6. Goods' attributes	1	5.6%	17	94.4%		0.0%	18
Total	45	38.4%	47	40.2%	25	21.2%	117

Table C-6 – Triggers and drivers (TD) involved in activating rebound mechanisms (RM). TD1.1: Revenues; TD1.2: Operational costs; TD1.3: Budget; TD1.4: Excess of supply of product; TD1.5: Excess of supply of process; TD2.1: Production time; TD2.2: Consumption time; TD2.3: Space savings; TD3.1: Awareness of consumption intensity; TD3.2: Convenience/Perception; TD3.3: Moral hazard; TD3.4: Moral credits; TD5.1: Reinvestment in innovation; TD5.2: Moral hazard; TD5.3: Moral credits; TD6.1: Substitutability of good; TD6.2: Substitutability of process; TD6.3: Time intensity to consume; TD6.4: Resource intensity of good; TD6.5: Resource intensity of process.

	TD. Economic/Financial						TD2. Physical constraints				TD3. Consumer choices					TD5. Company choices				TD6. Goods' attributes						Total
**Rebound mechanism (RM) \ Trigger or driver (TD)**	**1.1**	**1.2**	**1.3**	**1.4**	**1.5**	**TD1**	**2.1**	**2.2**	**2.3**	**TD2**	**3.1**	**3.2**	**3.3**	**3.4**	**TD3**	**5.1**	**5.2**	**5.3**	**TD5**	**6.1**	**6.2**	**6.3**	**6.4**	**6.5**	**TD6**	
RMA1.1: Income			x			1																				1
RMA1.2: Consumption time								x		1												x			1	2
RMA1.3: Motivational consumption											x	x			2											2
RMA1.4: Re-spending			x			1																	x		1	2
RMB2.1: Output	x	x				2																				2
RMB2.2: Production time							x			1																1
RMB2.3: Re-investment	x	x				2																		x	1	3
RMC2.4: Cost-dependent output	x	x				2																				2
RMA3.1: Substitution			x			1														x			x		2	3
RMA3.2: Motivational substitution											x	x			2					x			x		2	4
RMB3.3: Factor substitution	x	x				2														x	x		x	x	4	6
RMC3.4: Composition substitution	x	x				2														x	x		x	x	4	6
RMC3.5: Sectoral allocation	x	x				2														x	x		x	x	4	6
RMD4.1: Demand adjustment				x		1																				1
RMD4.3: Demand adjustment with investment adjustment				x		1																				1
RMB4.4: Re-design																										0
RMC4.5: Supply adjustment					x	1																				1
RMC4.6: Producer demand adjustment					x	1																				1
RMC4.7: Sector demand adjustment					x	1																				1
RMD5.1: Economies of scale	x	x	x			3																				3
RMD5.2: Market price	x	x				2																				2
RMD5.3: Labor income	x	x				2																				2
RMD5.5: New economic activity	x	x				2										x			1							3
Total	10	10	4	2	3	29	1	1	0	2	2	2	0	0	4	1	0	0	1	5	3	1	6	4	19	55
